# A Secure and Efficient Data Sharing and Searching Scheme in Wireless Sensor Networks

**DOI:** 10.3390/s19112583

**Published:** 2019-06-06

**Authors:** Binrui Zhu, Willy Susilo, Jing Qin, Fuchun Guo, Zhen Zhao, Jixin Ma

**Affiliations:** 1 School of Mathematics, Shandong University, Jinan 250100, China; zhubinrui1509889@163.com; 2 School of Computing and Information Technology, University of Wollongong, Wollongong 2522, Australia; wsusilo@uow.edu.au (W.S.); fuchun@uow.edu.au (F.G.); zz343@uowmail.edu.au (Z.Z.); 3 State Key Laboratory of Information Security, Institute of Information Engineering, Chinese Academy of Sciences, Beijing 100093, China; 4 Centre for Computer and Computational Science at School of Computing and Mathematical Sciences, University of Greenwich, London SE10 9LS, UK; j.ma@greenwich.ac.uk

**Keywords:** wireless sensor networks, cloud computing, Internet of Things, public key encryption with keyword search, off-line keyword guessing attack, on-line keyword guessing attack

## Abstract

Wireless sensor networks (WSN) generally utilize cloud computing to store and process sensing data in real time, namely, cloud-assisted WSN. However, the cloud-assisted WSN faces new security challenges, particularly outsourced data confidentiality. Data Encryption is a fundamental approach but it limits target data retrieval in massive encrypted data. Public key encryption with keyword search (PEKS) enables a data receiver to retrieve encrypted data containing some specific keyword in cloud-assisted WSN. However, the traditional PEKS schemes suffer from an inherent problem, namely, the keyword guessing attack (KGA). KGA includes off-line KGA and on-line KGA. To date, the existing literature on PEKS cannot simultaneously resist both off-line KGA and on-line KGA performed by an external adversary and an internal adversary. In this work, we propose a secure and efficient data sharing and searching scheme to address the aforementioned problem such that our scheme is secure against both off-line KGA and on-line KGA performed by external and internal adversaries. We would like to stress that our scheme simultaneously achieves document encryption/decryption and keyword search functions. We also prove our scheme achieves keyword security and document security. Furthermore, our scheme is more efficient than previous schemes by eliminating the pairing computation.

## 1. Introduction

Wireless sensor networks (WSN) and cloud computing have been widely deployed in daily life. WSN consists of small low-power sensors and lightweight mobile devices connected to the Internet [1,2]. These devices collect and exchange information in a variety of applications. Cloud computing has the advantages of unlimited capability in terms of both storage and computation. WSN is rapidly emerging, which is unprecedentedly driven by the assistance of cloud computing. As an emerging technology, WSN has utilized cloud computing to store and process data to reduce the burden of lightweight mobile devices.

More and more attention has been paid to using WSN technology as a crucial part of the Internet of Things (IoT) in various industries. IoT improves manufacturing efficiency and enables sustainable production [3,4,5,6,7]. As IoT and cloud-assisted WSN applications, enterprises and individuals have utilized cloud storage to complete the data storage and data sharing to reduce the burden of local storage.

**Figure 1:**
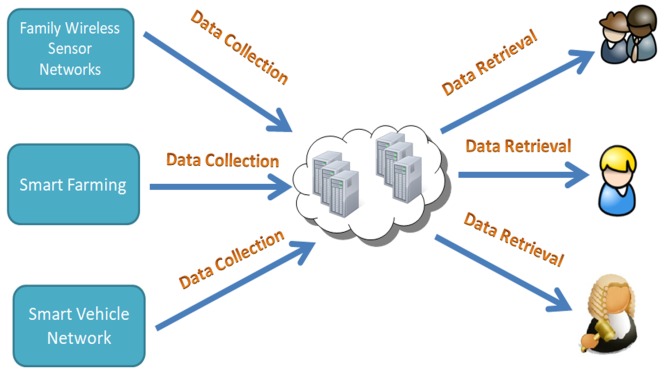
Functions of a cloud-assisted WSN.

As shown in Figure 1, the cloud-assisted WSN typical architecture. In this architecture, the cloud-assisted WSN system has powerful data processing capabilities and storage resources. The sensors implanted in the system collect data information and upload them to the cloud server by using a light mobile device. When the cloud-assisted WSN receives data, it stores and sends the data to relevant industry workers for utilization. In a specific practical scenario, such as a cloud-assisted medical system [8], the medical data documents are confidential to anyone except the patient and the chief physician. Consequently, the stored data should be guaranteed to be secure, since any information disclosure may result in serious consequences. Therefore, security requirements have become a key challenge in cloud-assisted WSN.

Security issues, such as users’ confidence that their data will remain secure with nobody able to modify or observe the contents, will remain the stumbling block that hinders the adoption of cloud-assisted WSN. Generally, users encrypt the data prior to uploading it to the cloud server for protecting data confidentiality. Unfortunately, this approach eliminates the data search services provided by modern search engines, which inevitably makes the effective data search function a challenging research problem. There are two trivial solutions to solve the search problem in encrypted documents. The first one is that the data receiver downloads the encrypted data locally, then decrypts the data and searches for the keyword at the local end. However, this method is impractical since it requires huge communication consumption and occupies a huge local storage space in the WSN. Another way is for the data receiver to send the authorization key to the cloud server which enables it to decrypt the encrypted documents in the cloud and to perform a search operation. However, this approach exposes data privacy to the cloud server and contradicts the original intention of data encryption. Focusing on the aforementioned problem, searchable encryption was proposed [9]. Searchable encryption enables a data receiver to authorize the cloud server to search in encrypted documents and returns the associated encrypted files, where the encrypted documents do not need to be decrypted.

Searchable encryption can be divided into symmetric searchable encryption (SSE) and public key encryption with keyword search (PEKS). In SSE, a shared key is required to achieve a data sharing function. PEKS [10] was proposed to eliminate the shared key in SSE. The general PEKS system includes three participants, that is, data senders, a data receiver and a cloud server. Data senders encrypt the data file and keywords index using the data receiver’s public key and then send ciphertexts to the cloud server. The data receiver uses its private key to generate a keyword trapdoor and transmits it to the cloud server. The cloud server uses the trapdoor to match the keyword ciphertext, if the keyword in the ciphertext and the keyword in the trapdoor are equal, it outputs equal; otherwise it outputs not equal.

**Figure 2:**
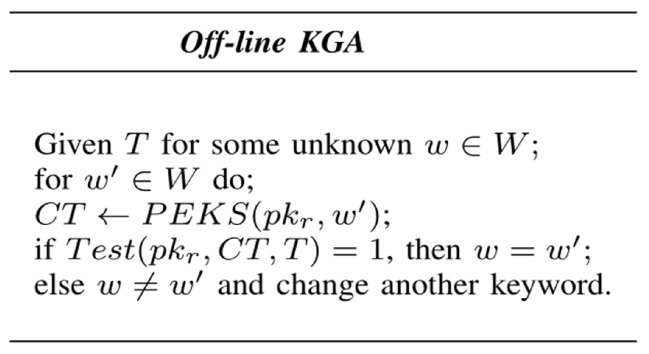
Off-line KGA.

Unfortunately, the traditional PEKS suffers from an inherent insecurity problem regarding trapdoor privacy. Anyone can use the data receiver’s public key to generate the valid keyword ciphertext. If the channel between the data receiver and cloud server is public, then the trapdoor is also open. If the adversary can execute the test algorithm, then it can verify whether or not the trapdoor and the ciphertext are matched. When they are well matched, the keyword in the trapdoor is equal to the keyword in the ciphertext; otherwise, the adversary can continue to guess another keyword until the correct keyword is found since the keyword space has a much smaller size. This kind of attack is called an off-line keyword guessing attack (off-line KGA), as shown in Figure 2. The off-line KGA is divided into an external adversary’s off-line KGA and an internal server adversary’s off-line KGA, according to which the adversary is an external adversary or an internal server adversary.

**Figure 3:**
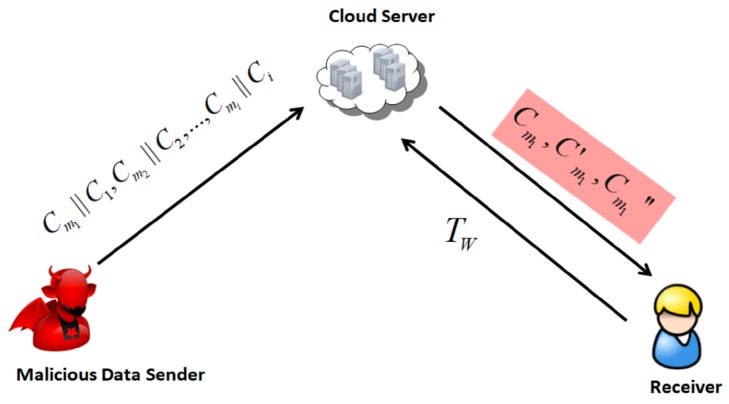
On-line KGA.

Besides, another inherent insecurity problem regarding trapdoor privacy exists in the traditional PEKS scheme. Since the keyword space has a much smaller size, a malicious data sender (including the external adversary) can generate a data file ciphertext and associated keyword ciphertext by guessing a keyword. If the channel between the data receiver and the cloud server is public, then the trapdoor to locate and return encrypted files is also open. After the cloud server performs the test matching operation, the related encrypted data files are returned. If the returned files have a encrypted data file generated by the malicious sender, the malicious data sender can determine the keyword associated with the encrypted data file, then the keyword in the trapdoor is also known to the malicious data sender. This kind of attack is called an on-line keyword guessing attack (on-line KGA), as shown in Figure 3. The difference between on-line KGA and off-line KGA mainly depends on whether the adversary attacks the scheme through the cloud server.

For both types of attacks, a trivial solution is that we need a secure channel to share the secret between the data receiver and data senders. A secure channel between cloud server and the data receiver can avoid the off-line KGA initiated by the external adversary and the on-line KGA. But the cost of building a secure channel prevents a Wi-Fi or 4G method from being utilized in the practical application. Moreover, for an internal server adversary, the data receiver and every data sender should share the secret in a secure channel against the off-line KGA initiated by the internal cloud adversary, while this method breaks the asymmetry property of PEKS. Therefore, it is significant and essential to resist both off-line KGA and on-line KGA performed by external and internal adversaries.

Considering a specific scenario: Personal Health Records (PHRs) are confidential documents to anyone except the patient and the chief physician. In order to protect patients’ PHR privacy, patients need to encrypt the PHR data prior to uploading it to the cloud server. We want to implement a search function, so a chief physician can search the PHR authorized information. We can use a PEKS scheme to solve the keyword search problem in encrypted PHR. However, the PEKS scheme suffers from an inherent problem, namely, the keyword guessing attack (KGA). In the process of searching, the adversary may obtain the keyword in the trapdoor, which exposes PHR data privacy to the adversary. Therefore, if we can design an efficient and secure data sharing and searching scheme to address the off-line KGA and on-line KGA problem, then data privacy will be guaranteed.

### 1.1. Our Contributions

In this paper, we study how to resist both off-line KGA and on-line KGA performed by external and internal adversaries in PEKS and propose a remedy to these problems. Specifically, our contributions are as follows:

1. We introduce a dating sharing and searching (DSS) frame that can effectively resist both off-line KGA and on-line KGA performed by an external adversary and an internal adversary. We also give a specific dual server DSS construction. The security of the scheme can achieve double ciphertext indistinguishability against the on-line KGA and indistinguishability against a chosen keyword attack (IND-CKA). We adopt the dual server method, which divides the cloud server into the forward server and backward server such that any single server cannot complete the test algorithm independently and any single server cannot get the correspondence between trapdoor and keyword ciphertext, therefore, the off-line KGA cannot be conducted successfully.

2. We add data file encryption/decryption to our scheme. In the traditional PEKS scheme, there is no algorithm for data file encryption/decryption. PEKS mainly focuses on the search process and omits the data file encryption/decryption process, which means there is only a keyword encryption algorithm in PEKS and it does not involve a data file encryption/decryption algorithm. However, in the actual application, a data file encryption/decryption is indispensable. The malicious data sender adversary may initiate an on-line KGA by observing the encrypted returned files. We adopt the re-encrypt technique, which the malicious data sender (including backward server) cannot get the correspondence between a trapdoor and encrypted data file, therefore, the on-line KGA cannot be conducted successfully.

3. Our scheme can simultaneously resist both off-line KGA and on-line KGA performed by external and internal adversaries. It does not require a secure channel and keeps the asymmetry property rather than a trivial solution. Compared to the previous schemes, our scheme also improves efficiency by eliminating the pairing computation and offers richer functionality by adding the data file encryption/decryption process.

Technical note: We choose PEKS as the starting point for the design of the scheme. For resisting KGA, we will discuss on-line KGA and off-line KGA. For an external adversary’s off-line KGA, the scheme generates a key pair for the cloud server to prevent the external adversary from launching an off-line KGA after eavesdropping the trapdoor through the public channel. What we need to point out here is to generate a key pair for the server it cannot entirely resist an external adversary’s off-line KGA. For example, Baek’s scheme has a fixed trapdoor. By comparing two bilinear pairs, the adversary can guess a keyword. We also need the trapdoor to satisfy the trapdoor indistinguishability to overcome this external adversary’s off-line KGA.

For an internal server adversary’s off-line KGA, we can divide the cloud server into two servers, which are the forward server and the backward server. Any single server cannot complete the test algorithm independently. Then, any single server cannot get the correspondence between the trapdoor and the keyword ciphertext, so the off-line KGA cannot be initiated. Therefore, our frame can resist off-line KGA performed by external and internal adversaries.

For on-line KGA, since the attack is initiated by observing the returned data files, we need to consider the data file encryption/decryption. We use the encryption scheme to provide data file encryption/decryption. The malicious data sender observes whether including the returned data file ciphertext is generated by itself to judge the keyword in eavesdropping on the trapdoor. Since the cloud server has strong computing power, we let the forward server perform double encryption for the data file ciphertext. In this way, the generated double ciphertext can satisfy the ciphertext indistinguishability for a malicious data sender, and therefore the malicious data sender adversary cannot initiate on-line KGA.

### 1.2. Related Works

In 2000, Song et al. first proposed an SSE scheme based on a symmetric cryptosystem [9]. Song et al.’s scheme can search any keyword in the ciphertext by word-by-word comparison to complete the keyword search function, therefore, the efficiency is low. Song et al.’s scheme suffers from statistical attacks and it cannot be proven secure. After Song et al.’s scheme, many researchers proposed SSE schemes [11,12]. The symmetric searchable encryption scheme can only be established under the symmetric cryptosystem, therefore, there is a problem of key distribution. In order to solve this problem, Boneh et al. proposed the first PEKS scheme based on the asymmetric cryptosystem in 2004 [10]. Boneh et al.’s scheme is transformed from identity-based encryption (IBE), which replaces the identity in the IBE with the keyword. Boneh et al.’s scheme needs a secure channel between the cloud server and the receiver for uploading the trapdoor. However, the cost of building a secure channel is expensive as is the connection between the receiver and cloud server through an insecure communication channel in IoT environment. In 2005, Abdalla et al. explored the conversion relationship between IBE and PEKS [13]. It is shown that an anonymous IBE scheme could be transformed into a PEKS scheme and it proposed the temporary keyword search scheme. Baek et al. proposed a PEKS scheme to remove the secure channel (dPEKS) [14]. In 2006, Baek et al. proposed a scheme combining a public key encryption (PKE) scheme and PEKS [15]. The scheme achieves the data file encryption/decryption function and keyword search function. Baek et al.’s scheme cannot resist off-line KGA, because the trapdoor in the Baek et al. scheme is fixed, the adversary can test each keyword through a bilinear pair to obtain the keyword in the trapdoor. Rhee et al. improved Baek et al.’s security model in 2009, which allows the adversary to obtain correspondence between the ciphertext and the trapdoor [16].

In 2010, Rhee et al. proposed a new dPEKS scheme [17]. The scheme proposed a new security definition, the trapdoor indistinguishability, and it is a sufficient condition for resisting the external adversary’s off-line KGA. In 2013, Fang et al. proposed a scheme that can resist the external adversary’s off-line KGA under the standard model [18]. Fang et al.’s scheme is the first dPEKS scheme to achieve the indistinguishability against a chosen keyword ciphertext attack that allows the adversary to initiate test query. Rhee et al’s two schemes and Fang et al’s scheme cannot resist internal server’s off-line KGA. In 2014, Chen et al. [19] proposed a generalized structure against on-line KGA. Chen et al.’s scheme [19] only satisfies the trapdoor security against on-line KGA and it also suffers from the off-line KGA. In 2016, Chen et al. proposed a two cloud server model [20] and any single server cannot complete the test operation so that it can resist the off-line KGA. However, in Chen et al.’s scheme [20], anyone who can generate a trapdoor and access the test query can create a security problem. It also cannot resist on-line KGA.

In 2016, Chen et al. proposed a joint scheme combining PKE and PEKS [21]. This scheme achieved the IND-CCA security and the indistinguishability against a chosen keyword ciphertext attack security but it could not resist both off-line and on-line KGA. In 2009, Tang et al. proposed a PEKS scheme for resisting off-line KGA [22]. Tang et al.’s method is to share the previously registered keywords between the receiver and every data sender. In 2017, Satio et al. proposed a PEKS scheme of designed-senders [23]. As a designed data sender, it needs to obtain the receiver’s authentication. Only the specified data sender can generate valid ciphertext and upload the shared encrypted data to the cloud server; therefore, the internal server adversary cannot generate valid ciphertext and cannot initiate the off-line KGA. In the same year, Huang et al. [24] and Jiang et al. [25] also used the idea of designed-senders. Only designed-senders can generate valid ciphertext so that it can resist the internal adversary’s off-line KGA. In 2018, Wu et al. proposed an off-line KGA scheme against an internal server adversary [26]. It is a method for sharing a secret between the data receiver and every sender. However, all the above five schemes have broken the asymmetry property of PEKS and cannot resist on-line KGA. Zhu et al. proposed a PEKS with a public verifiability scheme [27]. It achieves the public verifiability of the search results, but it cannot resist the internal server’s off-line KGA. Han et al. proposed a survey of keyword search schemes in recent years [28]. Many researchers also studied the keyword search problem [29,30].

After we finished our work, we found that Noroozi et al. concurrently presented a generalized PEKS structure against off-line KGA and on-line KGA for an external adversary [31]. It is a method to combine the PEKS with a designated server structure and the technique of re-randomizing ciphertexts. However, it is not enough for the PEKS scheme to resist this external adversary alone. The PEKS scheme still needs to resist an internal server adversary. In our work, we design a PEKS scheme that it simultaneously resists both external adversary and internal server adversary.

Noroozi et al. also considers that designing a PEKS scheme which is secure against off-line KGA and on-line KGA, even performed by the internal server adversary, remains a challenging problem.

We also found that this challenging problem still needs to be addressed. We designed a secure and efficient data sharing and searching (DSS) scheme against both off-line KGA and on-line KGA performed by external and internal adversaries.

### 1.3. Organization

The paper is organized as follows. The scheme definition and security model are described in Section 2. A secure and efficient data sharing and searching scheme against KGA (DSS against KGA) is proposed in Section 3. We analyze the security and efficiency of the proposed scheme in Section 3. The paper is concluded in Section 4.

## 2. Scheme Definition and Security Models

### 2.1. System Model

**Figure 4:**
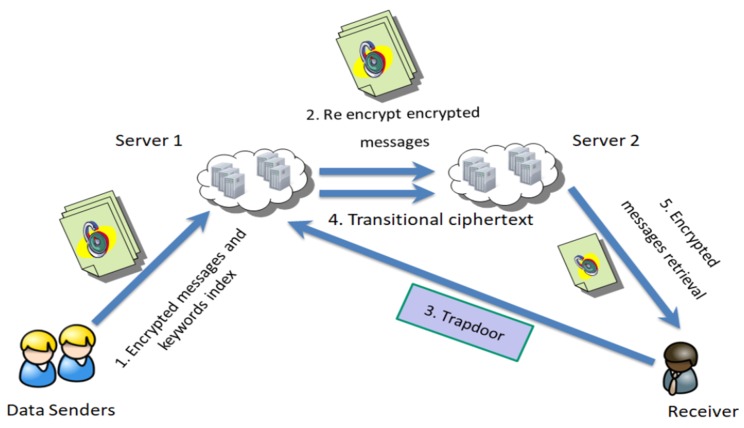
Dual server DSS against KGA model.

The model of the dual server DSS against KGA scheme (Dual server DSS against KGA model) that we proposed is shown in Figure 4. There are four participants in this model including data senders, a receiver, cloud sever 1 and cloud server 2. The workflow is as follows:

First of all, data senders encrypt the data file *M* using the data receiver’s public key $pk_{r}$ and encryption algorithm Enc to form a data file ciphertext $C_{1}$. Data senders also encrypt the corresponding keyword index using two servers’ public keys $pk_{s,1},pk_{s,2}$, the receiver’s public key $pk_{r}$ and the encryption algorithm peks to form keyword ciphertext $C_{2}$, then sends the ciphertext $\left(C_{1},C_{2}\right)$ to cloud server 1. Secondly, cloud server 1 generates the double ciphertext $C_{1}^{\prime }$ by re-encrypting the data file ciphertext $C_{1}$. Then, the data receiver uses its secret key $sk_{r}$ to generate a keyword trapdoor $T_{w}$ and transmits it to cloud server 1. Next, cloud server 1 uses the trapdoor $T_{w}$ and keyword ciphertext $C_{2}$ to compute the transitional ciphertext $C_{T}$, and sends the $C_{T}$ to cloud server 2. Afterwards, cloud server 2 outputs the matching result. If the keyword in the ciphertext and the keyword in the trapdoor are equal, cloud server 2 sends the relevant encrypted data file $C_{1}^{\prime }$ to the data receiver. In the final step, to obtain the message *M*, the receiver decrypts the data file’s double ciphertext $C_{1}^{\prime }$ using its secret key $sk_{r}$.

Although our scheme uses the re-encryption technique, its computational efficiency is almost equal to that of Noroozi et al.’s re-randomizing ciphertexts technique. Of course, the re-encryption technique can also be easily replaced with a re-randomizing ciphertexts technique in our work.

### 2.2. Algorithm Definitions

Before defining our algorithms, we define a notations Table 1 for the mathematical symbols in the whole paper.

More specifically, a scheme of DSS against KGA consists of the following algorithms:

(1) $\mathrm{sp}\leftarrow \mathrm{SysGen}(1^{k})$: on input a security parameter *k* and output a system parameter sp.
(2) KeyGen(sp):
  - $\left({\mathrm{pk}}_{s,1},{\mathrm{sk}}_{s,1}\right),\left({\mathrm{pk}}_{s,2},{\mathrm{sk}}_{s,2}\right)\leftarrow {\mathrm{KeyGen}}_{{\mathrm{server}}_{1,2}}\left(\mathrm{sp}\right)$: on input a system parameter sp and output two pairs of public and secret key $\left(pk_{s,1},sk_{s,1}\right),\left(pk_{s,2},sk_{s,2}\right)$ for the cloud server 1 and cloud server 2, separately.
  - $\left({\mathrm{pk}}_{r},{\mathrm{sk}}_{r}\right)\leftarrow {\mathrm{KeyGen}}_{\mathrm{receiver}}\left(\mathrm{sp}\right)$: on input a system parameter sp and output a pair of public and secret key $\left(pk_{r},sk_{r}\right)$ for the receiver.
(3) $\left(C_{1},C_{2}\right)\leftarrow \mathrm{PEKS}\left(\mathrm{sp},{\mathrm{pk}}_{s,1},{\mathrm{pk}}_{s,2},{\mathrm{pk}}_{r},w,M\right)$: on input a system parameter sp, the cloud server 1 public key $pk_{s,1}$, the cloud server 2 public key $pk_{s,2}$, the receiver public key $pk_{r}$, the keyword *w*, the message *M* and output the ciphertext $C_{1}=Enc\left(M,pk_{r},sp\right),C_{2}=peks\left(pk_{r},pk_{s,1},pk_{s,2},w,sp\right)$.
(4) $C_{1}^{\prime }\leftarrow \mathrm{ReEnc}\left(\mathrm{sp},{\mathrm{pk}}_{r},C_{1}\right)$: on input a system parameter sp, the receiver public key $pk_{r}$, the ciphertext $C_{1}$, and output the double ciphertext $C_{1}^{\prime }$.
(5) $T_{w}\leftarrow \mathrm{Trapdoor}\left(\mathrm{sp},{\mathrm{sk}}_{r},w,{\mathrm{pk}}_{s,1},{\mathrm{pk}}_{s,2},{\mathrm{pk}}_{r}\right)$: on input a system parameter sp, cloud server 1 public key $pk_{s,1}$, cloud server 2 public key $pk_{s,2}$, the receiver public key $pk_{r}$, the receiver secret key $sk_{r}$, the keyword *w*, and output the keyword search trapdoor $T_{w}$.
(6) $C_{1}^{\prime }\;\mathrm{or}⊥\leftarrow \mathrm{Test}\left(\mathrm{sp},T_{w},C_{2},{\mathrm{sk}}_{s,1},{\mathrm{sk}}_{s,2}\right)$: on input a system parameter sp, the cloud server 1 secret key $sk_{s,1}$, the cloud server 2 secret key $sk_{s,2}$, the keyword search trapdoor $T_{w}$, the ciphertext $\left(C_{1}^{\prime },C_{2}\right)$, and output ciphertext $C_{1}^{\prime }$ if the keyword search trapdoor $T_{w}$ matching the ciphertext $C_{2}$, and ⊥ otherwise. The matching process as follows:
  - ${\mathrm{Test}}_{1}\left(\mathrm{sp},T_{w},C_{2},{\mathrm{sk}}_{s,1}\right)\to C_{T}$: the cloud server 1 inputs the trapdoor $T_{w}$, the ciphertext $C_{2}$, the cloud server 1 secret key $sk_{s,1}$, the system parameter sp, and outputs the transitional ciphertext $C_{T}$.
  - ${\mathrm{Test}}_{2}\left(\mathrm{sp},C_{T},{\mathrm{sk}}_{s,2}\right)\to C_{1}^{\prime }\;\mathrm{or}\;⊥$: the cloud server 2 inputs the system parameter sp, the transitional ciphertext $C_{T}$, the cloud server 2 secret key $sk_{s,2}$. If the transitional ciphertext satisfies the condition, it outputs the double ciphertext $C_{1}^{\prime }$, and ⊥ otherwise.
(7) $M\leftarrow \mathrm{Dec}(\mathrm{sp},{\mathrm{sk}}_{r},C_{1}^{\prime })$: on input a system parameter sp, the receiver secret key $sk_{r}$, the ciphertext $C_{1}^{\prime }$ and output the message *M*.

### 2.3. Security Model

We define six security models, including the indistinguishability against a chosen keyword attack (IND-CKA 1) security model for cloud server 1, the IND-CKA 2 security model for cloud server 2, trapdoor indistinguishability against the off-line KGA (IND-Trapdoor 1) security model for cloud server 1, trapdoor indistinguishability against the off-line KGA (IND-Trapdoor 2) security model for cloud server 2, double ciphertext indistinguishability against the on-line KGA (IND-Double ciphertext) security model, transitional ciphertext indistinguishability against chosen keyword attack (IND-CKA 3) security model.

It should be noted that both cloud server 1 and cloud server 2 are “honest but curious” and they will not collude with each other. More specifically, the two servers strictly enforce the testing process of the algorithm but may be curious about the content of the keyword. It should be noted that these models implicitly define the security against external adversaries since the external adversary has less capability than the cloud server.

We define the keyword ciphertext’s semantic security. Any adversary cannot distinguish the challenge ciphertext unless the trapdoor is available. Formally, we define security model IND-CKA 1 and IND-CKA 2 played between a challenger B and adversary $A_{i},i=1,2$.

For the IND-CKA 1 security model, as the Table 2, the challenger B generates three key pairs $\left(pk_{s,1},sk_{s,1}\right),\left(pk_{s,2},sk_{s,2}\right),\left(pk_{r},sk_{r}\right)$. It sends public keys $pk_{s,1},pk_{s,2},pk_{r}$ and secret key $sk_{s,1}$ to the cloud server 1 adversary $A_{1}$. $A_{1}$ can access the trapdoor oracle $O_{1}\left(w\right)$ to get any keyword trapdoor $w_{i}$ and outputs two distinct challenge keywords and a message $\left(w_{0},w_{1},M^{*}\right)$, which $w_{b}\neq w_{i},b\in \left\{0,1\right\}$. The challenger B generates challenge PEKS ciphertext $\left(C_{1},C_{2,b}\right)$ of $\left(w_{b},M^{*}\right)$ with a random bit *b* and sends it to $A_{1}$. During the game, the adversary can adaptively continue to query trapdoor oracle $O_{1}\left(w\right)$ unless the challenge keywords $w_{0}$ and $w_{1}$. Finally, the adversary $A_{1}$ outputs $b^{\prime }$ as its guess.

For the IND-CKA 2 security model, as the Table 3, the game is similar to IND-CKA 1. We define security model IND-CKA 2 played between a challenger B and adversary $A_{2}$. We omit the details here. The definition is as follows:

**Definition** **1** **(IND-CKA).** *A scheme of DSS against the KGA is indistinguishable against a chosen keyword attack if no PPT adversaries $A_{1}$ can win game IND-CKA 1 and $A_{2}$ can win game IND-CKA 2 with a non-negligible advantage, where B is the challenger, $A_{1}$ is cloud server 1, $A_{2}$ is cloud server 2.*

*We define $A_{i}$ advantage as:*

$$Adv_{A_{i}}^{IND-CKA}=\left|\mathrm{Pr}\left[b=b^{\prime }\right]-1/2\right|,i\in \left\{1,2\right\}.$$

Next, we define the keyword trapdoor semantic security. Any adversary cannot distinguish the challenge trapdoor, that is to say, the challenge trapdoor does not reveal any information about the keyword. Formally, we define security model IND-Trapdoor 1 and IND-Trapdoor 2 played between a challenger B and adversary $A_{i},i=3,4$.

The IND-Trapdoor 1 and IND-Trapdoor 2 are similar to the IND-CKA 1. The adversary is given the challenge trapdoor instead of the PEKS challenge ciphertext. For the IND-Trapdoor 1 security model, as the Table 4, the challenger B generates three key pairs $\left(pk_{s,1},sk_{s,1}\right),\left(pk_{s,2},sk_{s,2}\right),\left(pk_{r},sk_{r}\right)$. It sends public keys $pk_{s,1},pk_{s,2},pk_{r}$ and secret key $sk_{s,1}$ to the cloud server 1 adversary $A_{3}$. $A_{3}$ can access the trapdoor oracle $O_{1}\left(w\right)$ to get any keyword trapdoor $w_{i}$ and outputs two distinct challenge keywords $\left(w_{0},w_{1}\right)$, which $w_{b}\neq w_{i},b\in \left\{0,1\right\}$. The challenger generates challenge trapdoor $T_{w_{b}}$ of $w_{b}$ with a random bit *b* and sends it to $A_{3}$. During the game, the adversary can adaptively continue to query trapdoor oracle $O_{1}\left(w\right)$ unless the challenge keywords $w_{0}$ and $w_{1}$. Finally, the adversary $A_{3}$ outputs $b^{\prime }$ as its guess.

For the IND-Trapdoor 2 security model, as the Table 5, the game is similar to IND-Trapdoor 1. We define security model IND-Trapdoor 2 played between a challenger B and adversary $A_{4}$. We omit the details here. The definition is as follows:

**Definition** **2** **(IND-Trapdoor).** *A scheme of DSS against the KGA is trapdoor indistinguishability against off-line KGA if no PPT adversaries $A_{3}$ can win the game IND-Trapdoor 1 and $A_{4}$ can win game IND-Trapdoor 2 with non-negligible advantage, where B is the challenger, $A_{3}$ is cloud server 1, $A_{4}$ is cloud server 2.*

*We define $A_{i}$ advantage as:*

$$Adv_{A_{i}}^{IND-Trapdoor}=\left|\mathrm{Pr}\left[b=b^{\prime }\right]-1/2\right|,i\in \left\{3,4\right\}.$$

After that, we define the double ciphertext semantic security. Any adversary cannot distinguish the challenge double ciphertext. Formally, we define the IND-Double ciphertext security model, as the Table 6. The IND-Double ciphertext is similar to the IND-CKA 1. The adversary outputs two distinct challenge ciphertext $\left(C_{1,0},C_{1,1}\right)$. The challenger generates double challenge ciphertext $C_{1,b}^{\prime }$ of $C_{1,b}$ with a random bit *b* and sends it to adversary. The adversary is given the challenge double ciphertext instead of the PEKS challenge ciphertext. Finally, the adversary outputs $b^{\prime }$ as its guess.

**Definition** **3** **(IND-Double** **ciphertext).** *A scheme of DSS against the KGA is double ciphertext indistinguishability against the on-line KGA if no PPT adversary $A_{5}$ can win the game IND-Double ciphertext with non-negligible advantage, where B is the challenger, $A_{5}$ is the malicious data sender (including the cloud server 2.*

*We define $A_{5}$ advantage as:*

$$Adv_{A_{5}}^{IND-Double\;ciphertext}=\left|\mathrm{Pr}\left[b=b^{\prime }\right]-1/2\right|.$$

Finally, we define the transitional ciphertext semantic security. Any adversary can not distinguish the challenge transitional ciphertext unless the trapdoor is available. Formally, we define security model IND-CKA 3, as the Table 7. The IND-CKA 3 is similar to the IND-CKA 1. The adversary is given the challenge transitional ciphertext instead of the PEKS challenge ciphertext. We omit the details here.

**Definition** **4** **(IND-CKA** **3).** *A scheme of DSS against the KGA is transitional ciphertext indistinguishability against chosen keyword attack if no PPT adversary $A_{6}$ can win the game IND-CKA 3 with non-negligible advantage, where B is the challenger and $A_{6}$ is an adversary (including the cloud server 2).*

*We define $A_{6}$ advantage as:*

$$Adv_{A_{6}}^{IND-CKA\;3}=\left|\mathrm{Pr}\left[\left(b_{1}^{\prime },b_{2}^{\prime }\right)=\left(b_{1},b_{2}\right)\right]-1/2\right|.$$

## 3. DSS against the KGA

In this section, we will propose a secure and efficient DSS scheme against the KGA. We use the Hashed Elgama scheme and a free channel PEKS scheme to construct the scheme.

### 3.1. Our Construction

Our instantiation of the proposed DSS general construction is described as follows: we add the receiver key generation algorithm, data file encryption/decryption algorithm and re-encryption algorithm. Meanwhile, we also eliminate the keyword and trapdoor security problem.

$\mathrm{SysGen}(1^{k})$: This algorithm inputs a security parameter $1^{k}$. It outputs a cyclic multiplicative group $G_{1}$ of prime order *p* and $g,g_{1},g_{2}\in G_{1}$, which *g* is generator of $G_{1}$. It selects three cryptographic hash functions $H_{1}:{\left\{0,1\right\}}^{*}\to {\left\{0,1\right\}}^{n},H_{2}:{\left\{0,1\right\}}^{*}\to G_{1},H_{3}:{\left\{0,1\right\}}^{*}\to {\left\{0,1\right\}}^{log_{2}^{p}+n}$. The algorithm outputs the system parameter
  $$sp=(G_{1},g,g_{1},g_{2},H_{1},H_{2},H_{3}).$$
KeyGen(sp):
  - ${\mathrm{KeyGen}}_{{\mathrm{server}}_{1,2}}\left(\mathrm{sp}\right)$: This algorithm inputs a system parameter sp. It chooses random number $\alpha_{1},\alpha_{2},\beta_{1},\beta_{2}$∈$Z_{p}^{*}$, and outputs the following $\left(pk_{s,1},sk_{s,1}\right)$ and $\left(pk_{s,2},sk_{s,2}\right)$ as the public/secret key pair of cloud server 1 and that of cloud server 2, separately.
    $$\left(pk_{s,1},sk_{s,1}\right)=\left(g_{1}^{\alpha_{1}}g_{2}^{\alpha_{2}},\left(\alpha_{1},\alpha_{2}\right)\right),$$
    $$\left(pk_{s,2},sk_{s,2}\right)=\left(g_{1}^{\beta_{1}}g_{2}^{\beta_{2}},\left(\beta_{1},\beta_{2}\right)\right).$$
  - ${\mathrm{KeyGen}}_{\mathrm{receiver}}\left(\mathrm{sp}\right)$: This algorithm inputs a system parameter sp. It chooses random number $c\in Z_{p}^{*}$ and outputs a pair of public and secret key $\left(pk_{r},sk_{r}\right)$ for the receiver,
    $$pk_{r}=g^{c},sk_{r}=c.$$
$\mathrm{PEKS}(\mathrm{sp},{\mathrm{pk}}_{s,1},{\mathrm{pk}}_{s,2},{\mathrm{pk}}_{r},w,M)$: This algorithm inputs a system parameter sp, the cloud server public key $pk_{s,1},pk_{s,2}$, the receiver public key $pk_{r}$, the keyword *w*, the message $M\in {\left\{0,1\right\}}^{n}$, and chooses random number $r_{0},r_{1}\in Z_{p}^{*}$. It outputs the message ciphertext $C_{1}=\left(C_{11},C_{12}\right)$, which
  $$C_{11}=g^{r_{0}},k=pk_{r}^{r_{0}},C_{12}=H_{1}\left(k\right)⊕M.$$
  It also outputs keyword ciphertext
  $$C_{2}=\left[A,B,C\right]=\left[g_{1}^{r_{1}},g_{2}^{r_{1}},pk_{s,1}^{r_{1}}\cdot pk_{s,2}^{r_{1}}\cdot pk_{r}\cdot H_{2}\left(w\right)\right].$$
$\mathrm{ReEnc}(\mathrm{sp},{\mathrm{pk}}_{r},C_{1})$: This algorithm inputs a system parameter sp, the receiver public key $pk_{r}$, the message ciphertext $C_{1}$. It chooses random number $r_{2}\in Z_{p}^{*}$ and outputs the double message ciphertext $C_{1}^{\prime }=\left(C_{11}^{\prime },C_{12}^{\prime }\right)$, which
  $$C_{11}^{\prime }=g^{r_{2}},k=pk_{r}^{r_{2}},C_{12}^{\prime }=H_{3}\left(k\right)⊕C_{11}∥C_{12}.$$
$\mathrm{Trapdoor}(\mathrm{sp},{\mathrm{pk}}_{s,1},{\mathrm{pk}}_{s,2},{\mathrm{pk}}_{r},{\mathrm{sk}}_{r},w)$: This algorithm inputs a system parameter sp, the cloud server public key $pk_{s,1},pk_{s,2}$, the receiver secret key $sk_{r}$, the keyword *w*, and chooses random number $r_{3}\in Z_{p}^{*}$. It outputs the keyword search trapdoor $T_{w}=\left[T_{1},T_{2},T_{3}\right],$
  $$T_{1}=g_{1}^{sk_{r}r_{3}},T_{2}=g_{2}^{sk_{r}r_{3}},$$
  $$T_{3}=pk_{s,1}^{sk_{r}r_{3}}\cdot pk_{s,2}^{sk_{r}r_{3}}\cdot pk_{r}^{-1}\cdot H_{2}^{-1}\left(w\right).$$
$\mathrm{Test}(\mathrm{sp},T_{w},C_{2},{\mathrm{sk}}_{s,1},{\mathrm{sk}}_{s,2})$:
  - ${\mathrm{Test}}_{1}\left(\mathrm{sp},T_{w},C_{2},{\mathrm{sk}}_{s,1}\right)\to C_{T}$: The cloud server 1 inputs the trapdoor $T_{w}$, the ciphertext $C_{2}$, the cloud server 1 secret key $sk_{s,1}$, the system parameter sp, and chooses random number $d\in Z_{p}^{*}$. It outputs the transitional ciphertext $C_{T}=\left(A^{*},B^{*},C^{*}\right)$, where
    $$T_{w}\cdot C_{2}=\left(C_{I,1},C_{I,2},C_{I,3}\right),$$
    $$C_{I,1}=T_{1}\cdot A,C_{I,2}=T_{2}\cdot B,C_{I,3}=T_{3}\cdot C,$$
    $$A^{*}=C_{I,1}^{d},B^{*}=C_{I,2}^{d},C^{*}={\left(\frac{C_{I,3}}{C_{I,1}^{\alpha_{1}}C_{I,2}^{\alpha_{2}}}\right)}^{d}.$$
  - ${\mathrm{Test}}_{2}\left(\mathrm{sp},C_{1}^{\prime },C_{T},{\mathrm{sk}}_{s,2}\right)\to C_{1}^{\prime }\;\mathrm{or}\;⊥$: The cloud server 2 inputs the system parameter sp, the transitional ciphertext $C_{T}$, the cloud server 2 secret key $sk_{s,2}$, and the double ciphertext $C_{1}^{\prime }$. It outputs $C_{1}^{\prime }$, if
    $$\frac{C^{*}}{A^{*\beta_{1}}B^{*\beta_{2}}}=1_{G},$$
    and ⊥ otherwise.
$\mathrm{Dec}(\mathrm{sp},{\mathrm{sk}}_{r},C_{1}^{\prime })$: This algorithm inputs a system parameter sp, the receiver secret key $sk_{r}$, the double message ciphertext $C_{1}^{\prime }$ and outputs the message
  $$C_{11}∥C_{12}=C_{1,2}^{\prime }⊕H_{3}\left(C_{1,1}^{\prime sk_{r}}\right),M=C_{1,2}⊕H_{1}\left(C_{1,1}^{sk_{r}}\right).$$
**Correctness:** When assuming the correctly generated ciphertext $C_{2}=\left[A,B,C\right]$ for $w_{i}$ with a correct trapdoor $T_{w}=\left(T_{1},T_{2},T_{3}\right)$. Then we can verify the equation for correctness if $w_{i}=w$ as follows:
  $$T_{w}C_{2}=\left(C_{I,1},C_{I,2},C_{I,3}\right),C_{I,1}=g_{1}^{r_{1}+cr_{3}},C_{I,2}=g_{2}^{r_{1}+cr_{3}},$$
  $$C_{I,3}={\left(g_{1}^{\alpha_{1}}g_{2}^{\alpha_{2}}\right)}^{r_{1}+cr_{3}}{\left(g_{1}^{\beta_{1}}g_{2}^{\beta_{2}}\right)}^{r_{1}+cr_{3}}H_{2}\left(w_{i}\right)H_{2}^{-1}\left(w\right).$$
  $$C_{T}=\left(A^{*},B^{*},C^{*}\right),A^{*}=g_{1}^{(r_{1}+cr_{3})d},B^{*}=g_{2}^{(r_{1}+cr_{3})d},$$
  $$C^{*}={\left({\left(g_{1}^{\beta_{1}}g_{2}^{\beta_{2}}\right)}^{\left(r_{1}+cr_{3}\right)}H_{2}\left(w_{i}\right)H_{2}^{-1}\left(w\right)\right)}^{d}.$$
  $$\frac{C^{*}}{A^{*\beta_{1}}B^{*\beta_{2}}}={\left(H_{2}\left(w_{i}\right)H_{2}^{-1}\left(w\right)\right)}^{d}=1_{G}$$

### 3.2. Proof

In the next theorems, we prove that our scheme satisfies indistinguishability against the chosen keyword attack and trapdoor indistinguishability against the off-line KGA, double ciphertext indistinguishability against the on-line KGA, transitional ciphertext indistinguishability against chosen keyword attack.

To prove our scheme security, we will use the widely accepted security reduction method. The security reduction is that if there is an adversary that can break our scheme, then the adversary can solve the hard mathematical problem. Mathematical hard problems are widely accepted and difficult to solve under existing computing ability. By the proof by contradiction, we can prove that our scheme is secure under the corresponding hard problem. By the security reduction, the scheme’s evaluation and validation are guaranteed. Related hard problems can be seen in Reference [32].

#### 3.2.1. Keyword Privacy

We prove that our scheme is secure following the Variant Decisional Diffie-Hellman Problem (Variant DDH) hard problem in Theorem 1 and Theorem 2.

Variant DDH Hard Problem [32]: Given the five tuple $\left(g,g^{a},g^{b},g^{ac},Z\right)$, $g,g^{a},g^{b},g^{ac},Z\in G_{1}$, where $G_{1}$ is a general cyclic group, all polynomial time algorithms decide the value $Z\overset{?}{=}g^{bc}$ is intractable.

**Theorem** **1.** *Under Variant DDH hard problem, the DSS scheme satisfies the keyword ciphertext indistinguishability in standard model, where the security reduction loss is 2.*

**Proof.**  (1) Suppose there is a cloud server 1 named adversary $A_{1}$ that can break our scheme in the IND-CKA 1 security model with advantage ε. In order to solve the Variant DDH hard problem, let’s construct a simulator B with a problem instance $\left(g_{1},g_{2},g_{1}^{a_{1}},g_{2}^{a_{2}}\right)$ over the cyclic group $G_{1}$. The simulation process is as follows:

**Setup.** Let $sp=(G_{1},g,g_{1},g_{2},H_{1},H_{2},H_{3})$. The simulator B chooses random elements $\alpha_{1},\alpha_{2},\beta_{1},\beta_{2},c\in Z_{p}^{*}$, and sets
  $$\left(pk_{s,1},sk_{s,1}\right)=\left(g_{1}^{\alpha_{1}}g_{2}^{\alpha_{2}},\left(\alpha_{1},\alpha_{2}\right)\right),$$
  $$\left(pk_{s,2},sk_{s,2}\right)=\left(g_{1}^{\beta_{1}}g_{2}^{\beta_{2}},\left(\beta_{1},\beta_{2}\right)\right),$$
  $$pk_{r}=g^{c},sk_{r}=c.$$
  The simulator B sends the public keys $pk_{s,2},pk_{r},pk_{s,1},sk_{s,1}$ to adversary $A_{1}$. B keeps the cloud 2’s secret key and receiver’s secret key for itself.
**Trapdoor Query.** The adversary $A_{1}$ can query $w_{i}$ to trapdoor oracle. The simulator chooses random number $r_{3}\in Z_{p}^{*}$ and outputs the keyword search trapdoor
  $$T_{w}=\left[T_{1},T_{2},T_{3}\right]=\left[g_{1}^{cr_{3}},g_{2}^{cr_{3}},pk_{s,1}^{cr_{3}}\cdot pk_{s,2}^{cr_{3}}\cdot pk_{r}^{-1}\cdot H_{2}^{-1}\left(w\right)\right].$$
  Therefore, the simulator completed the trapdoor query.
**Challenge.** The adversary $A_{1}$ gives two challenge words $w_{0}$,$w_{1}$ and the message $m^{*}$ to the simulator B, which $w_{b}\neq w_{i},b\in \left\{0,1\right\}$. The simulator returns a ciphertext $\left(C_{1},C_{2,b}\right)$. b∈{0,1} is randomly chosen. The simulator chooses random number $r_{0}\in Z_{p}^{*}$, and the ciphertext $\left(C_{1},C_{2,b}\right)$ is outputted as:
  $$C_{1}=\left(C_{11},C_{12}\right),C_{11}=g^{r_{0}},k=pk_{r}^{r_{0}},C_{12}=H_{1}\left(k\right)⊕m^{*},$$
  $$C_{2,b}=\left[g_{1}^{a_{1}},g_{2}^{a_{2}},{\left(g_{1}^{a_{1}}\right)}^{\alpha_{1}+\beta_{1}}{\left(g_{2}^{a_{2}}\right)}^{\alpha_{2}+\beta_{2}}g^{c}H_{2}\left(w_{b}\right)\right].$$
  Let $r_{1}=a_{1}.$ If $a_{1}=a_{2}$, we have
  $$C_{2,b}=\left[A,B,C\right]=\left[g_{1}^{r_{1}},g_{2}^{r_{1}},pk_{s,1}^{r_{1}}\cdot pk_{s,2}^{r_{1}}\cdot pk_{r}\cdot H_{2}\left(w_{b}\right)\right].$$
  Therefore, the challenge keyword ciphertext is a correct ciphertext.
**Trapdoor Query.** The adversary $A_{1}$ adaptively makes trapdoor query on $w_{i}$, $w_{i}\neq w_{0},w_{1}$. The simulator B computes trapdoor in the same way as above trapdoor query.
**Guess.** The adversary $A_{1}$ outputs $b^{\prime }$ as it’s guess.

Through the above description, we have completed the simulation process of the scheme and the simulation is correct, since the responses for the trapdoor query and challenge ciphertext are correct. Next, we will discuss the indistinguishable simulation. Random numbers include

$$\alpha_{1},\alpha_{2},\beta_{1},\beta_{2},c,r_{3},a_{1}=a_{2}.$$

All random numbers in simulation process are randomness. Therefore, the simulation with $a_{1}=a_{2}$ is indistinguishable, where the adversary wins the game with a probability of $1/2+\frac{\varepsilon }{2}$ as the breaking assumption.

When the $a_{1}\neq a_{2}$, in the following we show the analysis, the adversary wins the game with a maximum probability of 1/2.

Let $g_{2}=g_{1}^{z}$, the adversary knows

$$z,\alpha_{1},\alpha_{2},\alpha_{1}+z\alpha_{2},\beta_{1}+z\beta_{2},c$$

from the public key. The adversary knows

$$a_{1},a_{2},a_{1}\left(\alpha_{1}+\beta_{1}\right)+za_{2}\left(\alpha_{2}+\beta_{2}\right)+log_{g_{1}}^{H(w_{b})}.$$

from the challenge ciphertext.

Therefore, if $\alpha_{1},\alpha_{2},\beta_{1},\beta_{2}$ are known to the adversary, the adversary can guess the keyword $w_{b}$ correctly; else the the adversary cannot guess the keyword $w_{b}$ correctly. Since the adversary knows the $\alpha_{1},\alpha_{2},$ but not knows $\beta_{1},\beta_{2}$, the adversary has no advantage breaking the ciphertext. Therefore, the adversary wins the game with a probability of 1/2 by random guess.

Next, we will discuss the successful of the simulation, the simulator dose not abort the simulation in the trapdoor query and challenge phase. Therefore, the probability of successful simulation is $P_{s}=1.$ Therefore, the advantage of the Variant DDH hard problem is

$$\varepsilon_{R}=\left(1/2+\frac{\varepsilon }{2}-1/2\right)=\frac{\varepsilon }{2}.$$

(2) Suppose there is a cloud server 2 named adversary $A_{2}$ that can break our scheme in the IND-CKA 2 security model with advantage ε. In order to solve the Variant DDH hard problem, let’s construct a simulator B with a problem instance $\left(g_{1},g_{2},g_{1}^{a_{1}},g_{2}^{a_{2}}\right)$ over the cyclic group $G_{1}$. Simulation process is as follows:

**Setup.** Let $sp=(G_{1},g,g_{1},g_{2},H_{1},H_{2},H_{3})$. The simulator B chooses random elements $\alpha_{1},\alpha_{2},\beta_{1},\beta_{2},c\in Z_{p}^{*}$, and sets
  $$\left(pk_{s,1},sk_{s,1}\right)=\left(g_{1}^{\alpha_{1}}g_{2}^{\alpha_{2}},\left(\alpha_{1},\alpha_{2}\right)\right),$$
  $$\left(pk_{s,2},sk_{s,2}\right)=\left(g_{1}^{\beta_{1}}g_{2}^{\beta_{2}},\left(\beta_{1},\beta_{2}\right)\right),$$
  $$pk_{r}=g^{c},sk_{r}=c.$$
  The simulator B sends the public key $pk_{s,2},sk_{s,2},pk_{s,1},pk_{r}$ to adversary $A_{2}$. B keeps the cloud 1’s secret key and receiver’s secret key for itself.
**Trapdoor Query.** The adversary $A_{2}$ can query $w_{i}$ to trapdoor oracle. The simulator chooses random number $r_{3}\in Z_{p}^{*}$ and outputs the keyword search trapdoor
  $$T_{w}=\left[T_{1},T_{2},T_{3}\right]=\left[g_{1}^{cr_{3}},g_{2}^{cr_{3}},pk_{s,1}^{cr_{3}}\cdot pk_{s,2}^{cr_{3}}\cdot pk_{r}^{-1}\cdot H_{2}^{-1}\left(w\right)\right].$$
  Therefore, the simulator completed the trapdoor query.
**Challenge.** The adversary $A_{2}$ gives two challenge words $w_{0}$,$w_{1}$ and the message $m^{*}$ to the simulator B, which $w_{b}\neq w_{i},b\in \left\{0,1\right\}$. The simulator returns a ciphertext $\left(C_{1}^{\prime },C_{2,b}\right)$. b∈{0,1} is randomly chosen. The simulator chooses random number $r_{2}\in Z_{p}^{*}$, and the ciphertext $\left(C_{1}^{\prime },C_{2,b}\right)$ is outputted as:
  $$C_{1}^{\prime }=\left(C_{11}^{\prime },C_{12}^{\prime }\right),C_{11}^{\prime }=g^{r_{2}},k=pk_{r}^{r_{2}},C_{12}^{\prime }=H_{3}\left(k\right)⊕C_{1},$$
  which $C_{1}$ as the message encryption in the proposed scheme. It also outputs keyword ciphertext
  $$C_{2,b}=\left[g_{1}^{a_{1}},g_{2}^{a_{2}},{\left(g_{1}^{a_{1}}\right)}^{\alpha_{1}+\beta_{1}}{\left(g_{2}^{a_{2}}\right)}^{\alpha_{2}+\beta_{2}}g^{c}H_{2}\left(w_{b}\right)\right].$$
  Let $r=a_{1}.$ If $a_{1}=a_{2}$, we have
  $$C_{2,b}=\left[A,B,C\right]=\left[g_{1}^{r_{1}},g_{2}^{r_{1}},pk_{s,1}^{r_{1}}\cdot pk_{s,2}^{r_{1}}\cdot pk_{r}\cdot H_{2}\left(w_{b}\right)\right].$$
  Therefore, the challenge keyword ciphertext is correct.
**Trapdoor Query.** The adversary $A_{2}$ adaptively makes trapdoor query on $w_{i}$, $w_{i}\neq w_{0},w_{1}$. The simulator B computes trapdoor in the same way as above trapdoor query.
**Guess.** The adversary $A_{2}$ outputs $b^{\prime }$ as its guess.

As the entire indistinguishable analysis and probability analysis is similar to the above (1), we omit this process.

Therefore, the simulator solves the advantage of the Variant DDH hard problem

$$\varepsilon_{R}=\left(1/2+\frac{\varepsilon }{2}-1/2\right)=\frac{\varepsilon }{2}.$$

The Theorem 1 is proven.  □

Because the cloud server has more powerful attack capabilities than the external adversary, the scheme is also secure to external adversaries (including the receiver) in Theorem 1.

**Theorem** **2.** *Under Variant DDH hard problem, the DSS scheme satisfies trapdoor indistinguishability against the off-line KGA, where the security reduction loss is 2.*

**Proof.**  (1) Suppose there is a cloud server 1 named adversary $A_{3}$ that can break our scheme in IND-Trapdoor 1 security model with advantage ε. In order to solve the Variant DDH hard problem, let us construct a simulator B with a problem instance $\left(g_{1},g_{2},g_{1}^{a_{1}},g_{2}^{a_{2}}\right)$ over the cyclic group $G_{1}$. The simulation process is as follows:

**Setup.** Let $sp=(G_{1},g,g_{1},g_{2},H_{1},H_{2},H_{3})$. The simulator B chooses random elements $\alpha_{1},\alpha_{2},\beta_{1},\beta_{2},c\in Z_{p}^{*}$, and sets
  $$\left(pk_{s,1},sk_{s,1}\right)=\left(g_{1}^{\alpha_{1}}g_{2}^{\alpha_{2}},\left(\alpha_{1},\alpha_{2}\right)\right),$$
  $$\left(pk_{s,2},sk_{s,2}\right)=\left(g_{1}^{\beta_{1}}g_{2}^{\beta_{2}},\left(\beta_{1},\beta_{2}\right)\right),$$
  $$pk_{r}=g^{c},sk_{r}=c.$$
  The simulator B sends the public key $pk_{s,2},pk_{r},pk_{s,1},sk_{s,1}$ to adversary $A_{1}$. B keeps the cloud 2’s secret key and the receiver’s secret key for itself.
**Trapdoor Query.** The adversary $A_{3}$ can query $w_{i}$ to trapdoor oracle. The simulator chooses random number $r_{3}\in Z_{p}^{*}$ and outputs the keyword search trapdoor
  $$T_{w}=\left[T_{1},T_{2},T_{3}\right]=\left[g_{1}^{cr_{3}},g_{2}^{cr_{3}},pk_{s,1}^{cr_{3}}\cdot pk_{s,2}^{cr_{3}}\cdot pk_{r}^{-1}\cdot H_{2}^{-1}\left(w\right)\right].$$
  Therefore, the simulator completed the trapdoor query.
**Challenge.** The adversary $A_{3}$ gives two challenge words $w_{0},$ $w_{1}$ to the simulator B, which $w_{b}\neq w_{i},b\in \left\{0,1\right\}$. The simulator returns a challenge trapdoor $T_{w_{b}}$. b∈{0,1} is randomly chosen. The ciphertext $T_{w_{b}}$ is outputted as:
  $$T_{w_{b}}=\left[{\left(g_{1}^{a_{1}}\right)}^{c},{\left(g_{2}^{a_{2}}\right)}^{c},{\left({\left(g_{1}^{a_{1}}\right)}^{\alpha_{1}+\beta_{1}}{\left(g_{2}^{a_{2}}\right)}^{\alpha_{2}+\beta_{2}}\right)}^{c}g^{-c}H_{2}^{-1}\left(w_{b}\right)\right].$$
  Let $r_{3}=a_{1}.$ If $a_{1}=a_{2}$, we have
  $$T_{w}=\left[T_{1},T_{2},T_{3}\right]=\left[g_{1}^{cr_{3}},g_{2}^{cr_{3}},pk_{s,1}^{cr_{3}}\cdot pk_{s,2}^{cr_{3}}\cdot pk_{r}^{-1}\cdot H_{2}^{-1}\left(w_{b}\right)\right].$$
  Therefore, the challenge keyword trapdoor is a correct trapdoor.
**Trapdoor Query.** The adversary $A_{3}$ adaptively makes trapdoor query on $w_{i}$, $w_{i}\neq w_{0},w_{1}$. The simulator B computes trapdoor in the same way as above trapdoor query.
**Guess.** The adversary $A_{3}$ outputs $b^{\prime }$ as it’s guess.

As the entire indistinguishable analysis and probability analysis is similar to the above (1), we omit this process. Therefore, the simulator solving of the advantage of the Variant DDH hard problem is

$$\varepsilon_{R}=\left(1/2+\frac{\varepsilon }{2}-1/2\right)=\frac{\varepsilon }{2}.$$

(2) Suppose there is a cloud server 2 named adversary $A_{4}$ that can break our scheme in IND-Trapdoor 2 security model with advantage ε. The entire simulation process, solution algorithm and indistinguishable analysis is similar to the above (1), so we omit this process.

Therefore, the simulator solves the advantage of the Variant DDH hard problem

$$\varepsilon_{R}=\left(1/2+\frac{\varepsilon }{2}-1/2\right)=\frac{\varepsilon }{2}.$$

Therefore, the Theorem 2 is proven. □

Because the cloud server has more powerful attack capabilities than the external adversary, the scheme is also secure to external adversaries in Theorem 2.

We will prove that our scheme is secure following computational Diffie-Hellman (CDH) hard problem in Theorem 3.

CDH Hard Problem [15]: Given the three tuple $\left(g,g^{a},g^{b}\right)$, $g,g^{a},g^{b}\in G_{1}$, where $G_{1}$ is a general cyclic group of prime order *p*, all polynomial time algorithms compute the value $g^{ab}\in G_{1}$ is intractable.

**Theorem** **3.** *Under the CDH hard problem, the DSS scheme satisfies double ciphertext indistinguishability against on-line KGA in a random oracle model, where the security reduction loss is $\frac{1}{q_{H_{3}}}$.*

**Proof.**  Suppose there is an external adversary (including a cloud server 2) $A_{5}$ that can break our scheme in double ciphertext indistinguishability against on-line KGA security model with advantage ε. Suppose $H_{3}$ as a random oracle, in order to solve the CDH hard problem, let us construct simulator B with a problem instance $\left(g,g^{a},g^{b}\right)$ over the cyclic group $(G_{1},g,p).$ Our goal is to compute the value $g^{ab}$. The entire simulation process is as follows:

**Setup.** Let $sp=(G_{1},g,g_{1},g_{2},H_{1},H_{2},H_{3})$. The simulator B chooses random elements $\alpha_{1},\alpha_{2},\beta_{1},\beta_{2},c\in Z_{p}^{*}$, and sets
  $$\left(pk_{s,1},sk_{s,1}\right)=\left(g_{1}^{\alpha_{1}}g_{2}^{\alpha_{2}},\left(\alpha_{1},\alpha_{2}\right)\right),$$
  $$\left(pk_{s,2},sk_{s,2}\right)=\left(g_{1}^{\beta_{1}}g_{2}^{\beta_{2}},\left(\beta_{1},\beta_{2}\right)\right),$$
  $$pk_{r}=g^{a},sk_{r}=a.$$
  $sk_{r}$ is unknown to the simulator. The simulator B sends the public key $\left(pk_{s,2},sk_{s,2}\right),pk_{s,1},pk_{r}$ to adversary $A_{5}$ and keeps the cloud 1 secret key for itself.
**$H_{3}$-query**: The $H_{3}$ list is initially empty. The adversary $A_{5}$ can query $k_{i}\in G_{1}$ to $H_{3}$. If there exists a $\left(k_{i},X_{i}\right)$ in $H_{3}$ list, then the simulator B responds with $H_{3}\left(k_{i}\right)=X_{i}$; otherwise, the simulator B randomly chooses a value $X_{i}\in {\left\{0,1\right\}}^{log_{2}^{p}+n}$ and sets $H_{1}\left(k_{i}\right)=X_{i}$. It returns to the adversary $A_{5}$ and adds the value to $H_{3}$ list.
**Challenge.** The adversary $A_{5}$ gives two challenge ciphertext $C_{1,0},C_{1,1}$ to the simulator B. The simulator B returns ciphertext $C_{1,b_{0}}^{\prime }$. $b_{0}\in \left\{0,1\right\}$ is randomly chosen. The ciphertext $C_{1,b_{0}}^{\prime }$ is outputted as:
  $$C_{1,b_{0}}^{\prime }=\left[C_{1,1}^{\prime },C_{2,2}^{\prime }\right],C_{1,1}^{\prime }=g^{b},C_{2,2}^{\prime }=Z^{*},Z^{*}\in {\left\{0,1\right\}}^{d}.$$
  Define
  $$H_{3}\left(g^{ab}\right)=Z^{*}⊕C_{1,b_{0}},$$
**Guess.** The adversary $A_{5}$ outputs $b_{0}^{\prime }$ as its guess.

$Z^{*}$ is randomly chosen from ${\left\{0,1\right\}}^{d}.$ When the adversary does not query $g^{ab}$ to the random oracle, the challenge ciphertext is correct. Through the above description, we have completed the simulation process of the scheme and the simulation is correct. Next we will discuss the indistinguishable simulation. Random numbers include

$$X_{1},X_{2},...,X_{q_{H_{3}}},a,b,\alpha_{1},\alpha_{2},\beta_{1},\beta_{2},c.$$

Therefore, the simulation of the scheme is indistinguishable.

When the hash query is not a challenge hash query $g^{ab}$, the challenge message ciphertext is randomness, therefore, the adversary wins the game with a advantage 0.

The number of hash query is $q_{H_{3}}$. $A_{5}$ can break our scheme with advantage ε as the breaking assumption. Therefore, from the $H_{3}\left(k\right)$ list, we may find the correct challenge hash query $g^{ab}$. The probability of finding the correct challenge hash query is $P_{c}=\frac{1}{q_{H_{3}}}$. The simulator does not abort the simulation, therefore, the successful probability of the simulation is $P_{s}=1$.

The simulator solves the advantage of the CDH hard problem as

$$\varepsilon_{R}=\frac{\varepsilon }{q_{H_{3}}}.$$

Therefore, the Theorem 3 is proven. □

To secure against cloud server 1’s on-line KGA, we can let cloud server 2 use a re-encryption technique or a randomizing ciphertexts technique, we omit here the details.

**Theorem** **4.** *Under Variant DDH hard problem, the DSS scheme satisfies the transitional ciphertext indistinguishability in the standard model, where the security reduction loss is* 2.

**Proof.**  Suppose there is a cloud server 2 named adversary $A_{6}$ that can break our scheme in IND-CKA 3 security model with advantage ε. In order to solve the Variant DDH hard problem, let us construct a simulator B with a problem instance $\left(g_{1},g_{2},g_{1}^{a_{1}},g_{2}^{a_{2}}\right)$ over the cyclic group $G_{1}$. The simulation process is as follows:

**Setup.** Let $sp=(G_{1},g,g_{1},g_{2},H_{1},H_{2},H_{3})$. The simulator B chooses random elements $\alpha_{1},\alpha_{2},\beta_{1},\beta_{2},c\in Z_{p}^{*}$, and sets
  $$\left(pk_{s,1},sk_{s,1}\right)=\left(g_{1}^{\alpha_{1}}g_{2}^{\alpha_{2}},\left(\alpha_{1},\alpha_{2}\right)\right),$$
  $$\left(pk_{s,2},sk_{s,2}\right)=\left(g_{1}^{\beta_{1}}g_{2}^{\beta_{2}},\left(\beta_{1},\beta_{2}\right)\right),$$
  $$pk_{r}=g^{c},sk_{r}=c.$$
  The simulator B sends the public key $\left(pk_{s,2},sk_{s,2}\right),pk_{s,1},pk_{r}$ to adversary $A_{6}$. B keeps the cloud 1’s secret key and receiver’s secret key for itself.
**Trapdoor Query.** The adversary $A_{6}$ can query $w_{i}$ to trapdoor oracle. The simulator chooses random number $r_{3}^{\prime }\in Z_{p}^{*}$ and outputs the keyword search trapdoor
  $$T_{w}=\left[T_{1},T_{2},T_{3}\right]=\left[g_{1}^{cr_{3}^{\prime }},g_{2}^{cr_{3}^{\prime }},pk_{s,1}^{cr_{3}^{\prime }}\cdot pk_{s,2}^{cr_{3}^{\prime }}\cdot pk_{r}^{-1}\cdot H_{2}^{-1}\left(w\right)\right].$$
  The simulator completed the trapdoor query.
**Challenge.** The adversary $A_{6}$ gives two challenge words $w_{0},$ $w_{1}$ to the simulator B. $w_{b_{1}},w_{b_{2}}\neq w_{i},b_{1},b_{2}\in \left\{0,1\right\}$. The simulator generates a ciphertext $C_{2,b_{1}}$ and trapdoor $T_{w_{b_{2}}}$. $b_{1},b_{2}\in \left\{0,1\right\}$ are randomly chosen. The ciphertext $C_{2,b_{1}}$ and the trapdoor $T_{w_{b_{2}}}$ as:
  $$C_{2,b_{1}}=\left[g_{1}^{a_{1}},g_{2}^{a_{2}},{\left(g_{1}^{a_{1}}\right)}^{\alpha_{1}+\beta_{1}}{\left(g_{2}^{a_{2}}\right)}^{\alpha_{2}+\beta_{2}}g^{c}H_{2}\left(w_{b_{1}}\right)\right],$$
  $$T_{w_{b_{2}}}=\left[g_{1}^{cr_{3}},g_{2}^{cr_{3}},pk_{s,1}^{cr_{3}}\cdot pk_{s,2}^{cr_{3}}\cdot pk_{r}^{-1}\cdot H_{2}^{-1}\left(w_{b_{2}}\right)\right].$$
  Let $r_{1}=a_{1}.$ If $a_{1}=a_{2}$, we have
  $$C_{2,b_{1}}=\left[A,B,C\right]=\left[g_{1}^{r_{1}},g_{2}^{r_{1}},pk_{s,1}^{r_{1}}\cdot pk_{s,2}^{r_{1}}\cdot pk_{r}\cdot H_{2}\left(w_{b_{1}}\right)\right].$$
  Therefore, the transitional ciphertext is $C_{T^{*}}=\left(A^{*},B^{*},C^{*}\right)$, where
  $$T_{w_{b_{2}}}C_{2,b_{1}}=\left(C_{I,1},C_{I,2},C_{I,3}\right),A^{*}=C_{I,1}^{d},B^{*}=C_{I,2}^{d},$$
  $$C^{*}={\left(\left(g_{1}^{\left(a_{1}+cr_{3}\right)\beta_{1}}g_{2}^{\left(a_{2}+cr_{3}\right)\beta_{2}}\right)H_{2}\left(w_{b_{1}}\right)H_{2}^{-1}\left(w_{b_{2}}\right)\right)}^{d}.$$
  Therefore, the transitional ciphertext is a correct ciphertext.
**Trapdoor Query.** The adversary $A_{6}$ adaptively makes trapdoor query on $w_{i},$ $w_{i}\neq w_{0},w_{1}$. The simulator B computes the trapdoor in the same way as above trapdoor query.
**Guess.** The adversary $A_{6}$ outputs $\left(b_{1}^{\prime },b_{2}^{\prime }\right)$ as it’s guess.

When $a_{1}=a_{2},$ the indistinguishable analysis is similar to Theorem 1, we omit this process.

When the $a_{1}\neq a_{2}$, in the following we show the analysis, the adversary wins the game with probability of 1/2.

Let $g_{2}=g_{1}^{z}$, the adversary knows $z,\beta_{1},\beta_{2},\alpha_{1}+z\alpha_{2},\beta_{1}+z\beta_{2},c$ from the public key. The adversary knows

$$\left(a_{1}+cr_{3}\right)d,\left(a_{2}+cr_{3}\right)d,$$

$$\left(a_{1}+cr_{3}\right)d\beta_{1}+z\left(a_{2}+cr_{3}\right)d\beta_{2}+log_{g_{1}}^{{\left(H\left(w_{b_{1}}\right)H_{2}^{-1}\left(w_{b_{2}}\right)\right)}^{d}}.$$

from the challenge ciphertext.

Therefore, if the *d* is known, the adversary will guess keywords $\left(w_{b_{1}},w_{b_{2}}\right)$ correctly; else the

$$\left(a_{1}+cr_{3}\right)d\beta_{1}+z\left(a_{2}+cr_{3}\right)d\beta_{2}+log_{g_{1}}^{{\left(H\left(w_{b_{1}}\right)H_{2}^{-1}\left(w_{b_{2}}\right)\right)}^{d}}$$

hides the $\left(w_{b_{1}},w_{b_{2}}\right)$. Since the adversary does not know the *d*, it also has no advantage in breaking the ciphertext.

Next, the successful simulation probability is $P_{s}=1.$ The simulator solves the advantage of the Variant DDH hard problem as

$$\varepsilon_{R}=\left(1/2+\frac{\varepsilon }{2}-1/2\right)=\frac{\varepsilon }{2}.$$

Therefore, the Theorem 4 is proven. □

#### 3.2.2. Message Privacy

Regarding the security of the message, the proof is similar to Theorem 3 and is based on the CDH hard problem in the random oracle model. Since it is too similar, we omit the proof here.

### 3.3. Analysis and Comparisons

We use Table 8 and Table 9 to show two comparisons between our scheme and previous schemes. In this section, the word abbreviation Trap Ind, MCiph Ind, KCiph Ind, In-off-line KGA, Ex-off-line KGA, on-line KGA, MCiph, KCiph to denote trapdoor indistinguishability, message ciphertext indistinguishability, keyword ciphertext indistinguishability, off-line keyword guessing attack for internal attacker, off-line keyword guessing attack for external attacker, on-line keyword guessing attack, message ciphertext, keyword ciphertext. We use $e,E_{1},E_{1}^{\prime },E_{2}^{\prime },h,I,PM$ to denote a pairing operation, an exponentiation operation in cyclic multiplicative group $G_{1}$, an exponentiation operation in $G_{1}^{\prime }$ from paring, an exponentiation operation in $G_{T}^{\prime }$ from paring, a hash operation maps a string to an element of cyclic group, an inverse operation, a multiplication in $G_{1}^{\prime }$ from paring. We ignore other hash operations and multiplication.

**Figure 5:**
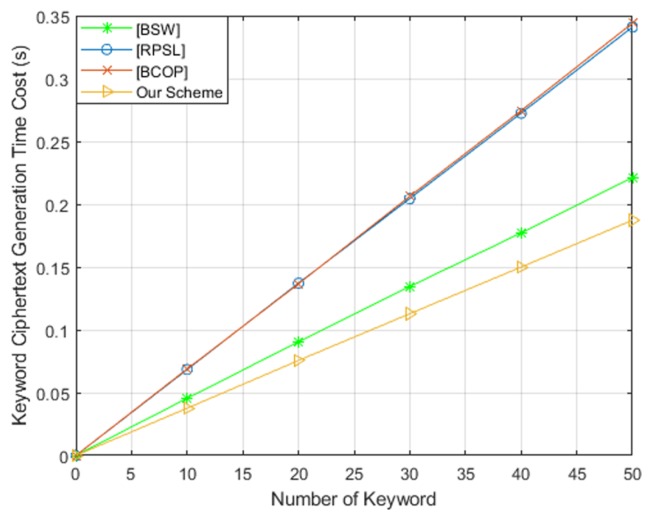
Computation cost of keyword ciphertext generation.

**Figure 6:**
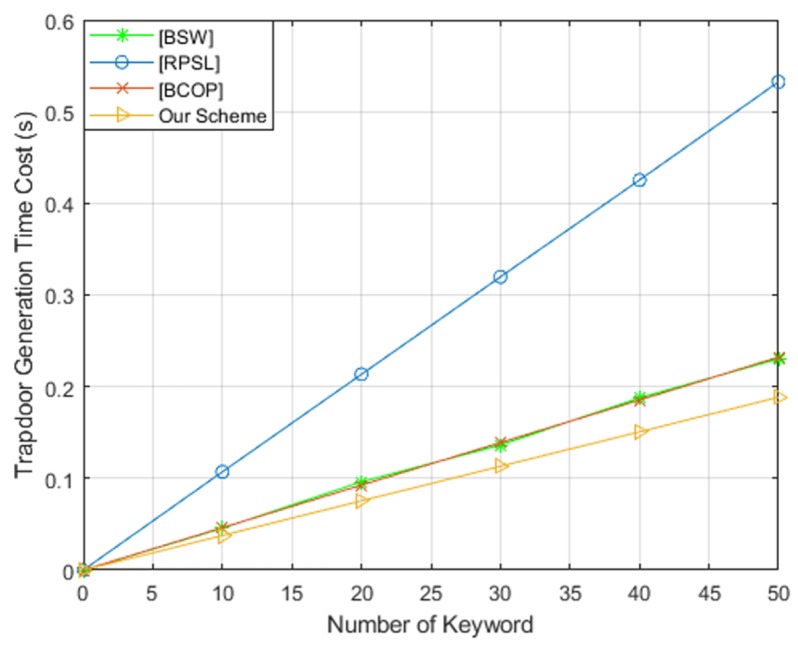
Computation cost of trapdoor generation.

**Figure 7:**
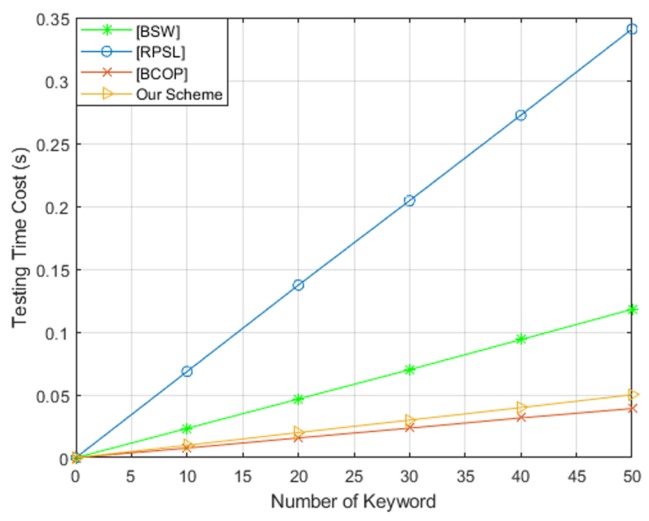
Computation cost of test algorithm.

To evaluate the efficiency of our scheme, we implemented theses schemes on a Core(TM) i7-6500U CPU at 2.50GHz and 4GB RAM (3.89GB is available) running Ubuntu 18.04. We used a Type-A pairing elliptic curve and implemented in the PBC library. For these four schemes, we tested the running time of keyword ciphertext generation, trapdoor generation and test algorithms, respectively. The comparison results are shown in Figure 5, Figure 6 and Figure 7. From these three figures, we found that our scheme is the most efficient in terms of keyword ciphertext generation and trapdoor generation algorithms. Although our scheme’s test algorithm is slightly less computationally efficient than BCOP [10] scheme. However, in comparison with other PEKS schemes, our efficiency remains high by eliminating the pairing computation and exponentiation operation in $G_{1}^{\prime }$. Furthermore, our scheme also offers a stronger security guarantee for keyword security.

### 3.4. Research Method

In our paper, we researched the trapdoor security problem in a WSN environment in the following way, which is motivation ⇒ application scenario ⇒ technical rote ⇒ frame architecture ⇒ security model ⇒ concrete construction ⇒ security reduction ⇒ efficiency analysis and comparisons.

## 4. Conclusions

The combination of cloud computing and WSN provides a promising solution to handle massive data. Data security requirements have become a key challenge in cloud-assisted WSN. To address limitations inherent in data security problems, in this paper, we defined a secure and efficient DSS scheme that can resist both off-line KGA and on-line KGA performed by external adversary and internal adversary, and we proposed a specific construction. This construction can simultaneously resist both on-line KGA and off-line KGA in cloud-assisted WSN. Our scheme not only realizes the keyword search function in the cloud but also implements the data files encryption/decryption function. The performance analysis shows the computation overhead at lightweight mobile devices is significantly reduced. We also formally proved that our schemes are provably secure.

## Figures and Tables

**Table 1:** Notations.

Notation	Description
sp	System parameter
$pk_{s,1},sk_{s,1}$	Public/secret key of the cloud server 1
$pk_{s,2},sk_{s,2}$	Public/secret key of the cloud server 2
$pk_{r},sk_{r}$	Public/secret key of the receiver
*w*	Keyword
*m*	Message
Enc(m)	Encryption algorithm Enc for the data *m*
peks(m)	Encryption algorithm peks for the keyword *w*
$C_{1}$	Message ciphertext
$C_{2}$	Searchable ciphertext for keyword
$C_{1}^{\prime }$	Double message ciphertext
$T_{w}$	Trapdoor for keyword *w*
$C_{T}$	The transitional ciphertext
A	Adversary
B	Challenger or simulator
O(w)	Trapdoor oracle for the keyword *w*

**Table 2:** IND-CKA 1.

*Game IND-CKA 1 ${\mathrm{Exp}}_{A_{1}}^{\mathrm{CKA}}\left[\mathrm{DSS}\right]$*
Kset⟵ϕ
$\left(pk_{s,1},sk_{s,1},pk_{s,2},sk_{s,2},pk_{r},sk_{r}\right)⟵KeyGen\left(sp\right)$;
$\left(w_{0},w_{1},M^{*}\right)⟵A_{1}^{O}\left(sp,\left(pk_{s,1},sk_{s,1}\right),pk_{s,2},pk_{r}\right)$;
$\left(C_{1},C_{2,b}\right)⟵B\left(M^{*},w_{b},pk_{s,1},pk_{s,2},pk_{r},b\in \left\{0,1\right\}\right)$;
$b^{\prime }⟵A_{1}^{O}\left(C_{1},C_{2,b},guess\right)$;
if $\{w_{0},w_{1}\}⋂Kset=\phi$, then return 1, if $b^{\prime }=b$;
else return 0.
Oracle O(w):
Kset=Kset⋃{w}, $T_{w}⟵O\left(pk_{s,1},pk_{s,2},pk_{r},sk_{r},w\right)$;
return $\left\{T_{w}\right\}$

**Table 3:** IND-CKA 2.

*Game IND-CKA 2 ${\mathrm{Exp}}_{A_{2}}^{\mathrm{CKA}}\left[\mathrm{DSS}\right]$*
Kset⟵ϕ
$\left(pk_{s,1},sk_{s,1},pk_{s,2},sk_{s,2},pk_{r},sk_{r}\right)⟵KeyGen\left(sp\right)$;
$\left(w_{0},w_{1},M^{*}\right)⟵A_{2}^{O}\left(sp,\left(pk_{s,2},sk_{s,2}\right),pk_{s,1},pk_{r}\right)$;
$\left(C_{1}^{\prime },C_{2,b}\right)⟵B\left(M^{*},w_{b},pk_{s,1},pk_{s,2},pk_{r},b\in \left\{0,1\right\}\right)$;
$b^{\prime }⟵A_{2}^{O}\left(C_{1}^{\prime },C_{2,b},guess\right)$;
if $\{w_{0},w_{1}\}⋂Kset=\phi$, then return 1, if $b^{\prime }=b$;
else return 0.
Oracle O(w):
Kset=Kset⋃{w}, $T_{w}⟵O\left(pk_{s,1},pk_{s,2},pk_{r},sk_{r},w\right)$;
return $\left\{T_{w}\right\}$

**Table 4:** IND-Trapdoor 1.

*Game IND-Trapdoor 1 ${\mathrm{Exp}}_{A_{3}}^{\mathrm{off}-\mathrm{line}\;\mathrm{KGA}}\left[\mathrm{DSS}\right]$*
Kset⟵ϕ
$\left(pk_{s,1},sk_{s,1},pk_{s,2},sk_{s,2},pk_{r},sk_{r}\right)⟵KeyGen\left(sp\right)$;
$\left(w_{0},w_{1}\right)⟵A_{3}^{O}\left(sp,\left(pk_{s,1},sk_{s,1}\right),pk_{s,2},pk_{r}\right)$;
$T_{b}⟵B\left(w_{b},pk_{s,1},pk_{s,2},pk_{r},sk_{r},b\in \left\{0,1\right\}\right)$;
$b^{\prime }⟵A_{3}^{O}\left(T_{b},guess\right)$;
if $\{w_{0},w_{1}\}⋂Kset=\phi$, then return 1, if $b^{\prime }=b$;
else return 0.
Oracle O(w):
Kset=Kset⋃{w}, $T_{w}⟵O\left(pk_{s,1},pk_{s,2},pk_{r},sk_{r},w\right)$;
return $\left\{T_{w}\right\}$

**Table 5:** IND-Trapdoor 2.

*Game IND-Trapdoor 2 ${\mathrm{Exp}}_{A_{4}}^{\mathrm{off}-\mathrm{line}\;\mathrm{KGA}}\left[\mathrm{DSS}\right]$*
Kset⟵ϕ
$\left(pk_{s,1},sk_{s,1},pk_{s,2},sk_{s,2},pk_{r},sk_{r}\right)⟵KeyGen\left(sp\right)$;
$\left(w_{0},w_{1}\right)⟵A_{4}^{O}\left(sp,pk_{s,1},\left(pk_{s,2},sk_{s,2}\right),pk_{r}\right)$;
$T_{b}⟵B\left(w_{b},pk_{s,1},pk_{s,2},pk_{r},sk_{r},b\in \left\{0,1\right\}\right)$;
$b^{\prime }⟵A_{4}^{O}\left(T_{b},guess\right)$;
if $\{w_{0},w_{1}\}⋂Kset=\phi$, then return 1, if $b^{\prime }=b$;
else return 0.
Oracle O(w):
Kset=Kset⋃{w}, $T_{w}⟵O\left(pk_{s,1},pk_{s,2},pk_{r},sk_{r},w\right)$;
return $\left\{T_{w}\right\}$

**Table 6:** IND-Double ciphertext.

*Game IND-Double ciphertext ${\mathrm{Exp}}_{A_{5}}^{\mathrm{Online}-\mathrm{KGA}}\left[\mathrm{DSS}\right]$*
$\left(pk_{s,1},sk_{s,1},pk_{s,2},sk_{s,2},pk_{r},sk_{r}\right)⟵KeyGen\left(sp\right)$;
$\left(C_{1,0},C_{1,1}\right)⟵A_{5}\left(sp,pk_{s,1},\left(pk_{s,2},sk_{s,2}\right),pk_{r}\right)$;
$C_{1,b}^{\prime }⟵B\left(C_{1,b},pk_{s,1},pk_{s,2},pk_{r},b\in \left\{0,1\right\}\right)$;
$b^{\prime }⟵A_{5}\left(C_{1,b}^{\prime },guess\right)$;
Then return 1, if $b^{\prime }=b$; else return 0.

**Table 7:** IND-CKA 3.

*Game IND-CKA 3 ${\mathrm{Exp}}_{A_{6}}^{\mathrm{CKA}}\left[\mathrm{DSS}\right]$*
Kset⟵ϕ
$\left(pk_{s,1},sk_{s,1},pk_{s,2},sk_{s,2},pk_{r},sk_{r}\right)⟵KeyGen\left(sp\right)$;
$\left(w_{0},w_{1}\right)⟵A_{6}^{O}\left(sp,\left(pk_{s,2},sk_{s,2}\right),pk_{s,1},pk_{r}\right)$;
$C_{2,b_{1}}⟵B\left(w_{b_{1}},pk_{s,1},pk_{s,2},pk_{r},b_{1}\in \left\{0,1\right\}\right)$;
$T_{w_{b_{2}}}⟵B\left(w_{b_{2}},pk_{s,1},pk_{s,2},pk_{r},sk_{r},b_{2}\in \left\{0,1\right\}\right)$;
$C_{T}⟵B\left(C_{2,b_{1}},T_{w_{b_{2}}},pk_{s,1},sk_{s,1},pk_{s,2},pk_{r}\right)$;
$\left(b_{1}^{\prime },b_{2}^{\prime }\right)⟵A_{6}^{O}\left(C_{T},guess\right)$;
if $\{w_{0},w_{1}\}⋂Kset=\phi$, then return 1, if $\left(b_{1}^{\prime },b_{2}^{\prime }\right)=\left(b_{1},b_{2}\right)$;
else return 0.
Oracle O(w):
Kset=Kset⋃{w}, $T_{w}⟵O\left(pk_{s,1},pk_{s,2},pk_{r},sk_{r},w\right)$;
return $\left\{T_{w}\right\}$

**Table 8:** Computation comparison.

	BCOP [10]	BSW [14]	RPSL [17]	Our
MCiph	-	-	-	$2E_{1}$
KCiph	$2E_{1}^{\prime }+h+e$	$E_{1}^{\prime }+E_{2}^{\prime }+h+2e$	$2E_{1}^{\prime }+h+e$	$4E_{1}+h$
ReEnc	-	-	-	$2E_{1}$
Trapdoor	$E_{1}^{\prime }+h$	$E_{1}^{\prime }+h$	$3E_{1}^{\prime }+2h+I+PM$	$4E_{1}+h+2I$
Test	*e*	$E_{1}^{\prime }+e+PM$	$2E_{1}^{\prime }+h+e+PM$	$7E_{1}$
Dec	-	-	-	$2E_{1}$

**Table 9:** Security comparison.

	BCOP [10]	BSW [14]	RPSL [17]	Our
Trap Ind	NO	NO	YES	YES
MCiph Ind	-	-	-	YES
KCiph Ind	YES	YES	YES	YES
In-off-line KGA	NO	NO	NO	YES
Ex-off-line KGA	NO	NO	YES	YES
on-line KGA	NO	NO	NO	YES
